# Prize Essay on Nostrums

**Published:** 1850-07

**Authors:** 


### PRIZE ESSAY ON NOSTRUMS.

We copy the following from the July number of the New York Journal of Medicine:

*Prize Essay on Nostrums.*—The committee of the New York State

Medical Society, on prize dissertations, reported at its last session the reception of one essay on the subject for which the prize was offered last year. This being not in accordance with the spirit and requirements of the resolution under which it was offered, they recommended the passage of the following resolution, which was adopted:

*Resolved*, That a prize of $20 be offered for the best essay "on the pernicious influences of nostrums and secret remedies on the health and morals of the community." Said essay to consist of not less than 16 pages, or more than 20 of the Transactions, to be adapted for popular, rather than professional instruction. The essays to be transmitted to the secretary of the society, before the 1st of January, 1851.

JAMES McNAUGHTON, Chairman.

The subject, on which this premium is offered is one of great moment to the medical profession, but of infinitely greater importance to the public. Each nostrum manufacturer takes the sentiment of John Wilkes for his guide—"the public is a goose and I have as much right to pluck a feather as others," and between lying advertisements, and the certificates of clergymen, which are always easily procured, the swindling quack plucks his bird terribly. And the ebbing and flowing of the tides are not more marked than the fashions of nostrums. A few years ago, Morrison's Hygiene pills overshadowed England and America. Moat, struck with the success in this country, attempted an independent standard for himself, and the press was called into requisition to magnify the merits of the rival claimants to the only true and genuine Hygiene pill. We recollect the case of an agent who lost his wife despite of the multiform virtues of the universal pill, and being somewhat alarmed for the fate of his pill-vending, he took good care to announce in the obituary notice of his wife, that she died, notwithstanding the skill and attention of two eminent physicians, whose names were duly paraded in the post mortem announcement. The inconsolable widower was determined that his mishap should nor stop the sale of the universal pills.

In the midst of the quarrels of Morrison and Moat, that arch-humbug and quack, Benjamin Brandreth, began to loom forth on the vision of an afflicted public, and, by dint of eternal falsehood, he succeeded in moving the bowels of the people. Stars of lesser magnitude rolled in the nebulæ of his round of purgation, and the newspapers were darkened with the wings of vultures hastening to their orgies. Doctors were extemporaneously manufactured for the manufacture of certificates, and clergymen told, in pious terms, what the various medicines had done for them, "under the blessing of God."

The next great evil is the "Domestic Medicine Book," an imposture and swindle that commands the aid of physicians. These books are sent forth to the world under the auspices of the most respectable names in the profession, and letters are published from these gentlemen, which declare that the writers have examined the book, and found it very good. The aim of the book is an insult to common sense. It assumes that by descriptions of diseases, in plain English, any one may be able to detect the particular disease in hand, and prescribe for it, without regard to idiosyncracy, age, sex, temperament, peculiarity, or the probable mistake in diagnosis! And physicians, are found capable of lending their names to this humbug! We would as soon be found certifying to the virtues of Swaim's Panacea, John Bull's Sarsaparilla. Brandreth's Pills, or Number Six. The object of the nostrum maker, and of the author of "The Domestic Medicine" is precisely the same, and he that can aid one, should have no kind of hesitation in being trumpeter for the other.

There is a serious evil connected with the swilling of sarsaparilla which should command the attention of medical men. Nearly all the preparations in market contain iodide of potash, and derive all their power from it. Those who swallow sarsaparilla are almost sure to dabble in other medicines, blue pill and calomel especially. Now medical men know the great danger in using the preparations of iodine and mercury together. They form an iodide of mercury which is an active poison. This is one of the evils of nostrums.

We are glad that the New York State Medical Society has taken up this subject, and we hope the premium offered, though small, will call into being a good essay. B.

